# Atrio-esophageal fistula clinically presented as pericardial-esophageal fistula

**DOI:** 10.1007/s10840-020-00922-8

**Published:** 2021-01-12

**Authors:** Nándor Szegedi, Imre Ferenc Suhai, Péter Perge, Zoltán Salló, István Hartyánszky, Béla Merkely, László Gellér

**Affiliations:** Heart and Vascular Center, Semmelweis University, Városmajor Street 68., Budapest, 1122 Hungary

**Keywords:** Atrio-esophageal fistula, Pericardial-esophageal fistula, Atrial fibrillation, Ablation, Fatal complication

**Fig. 1:**
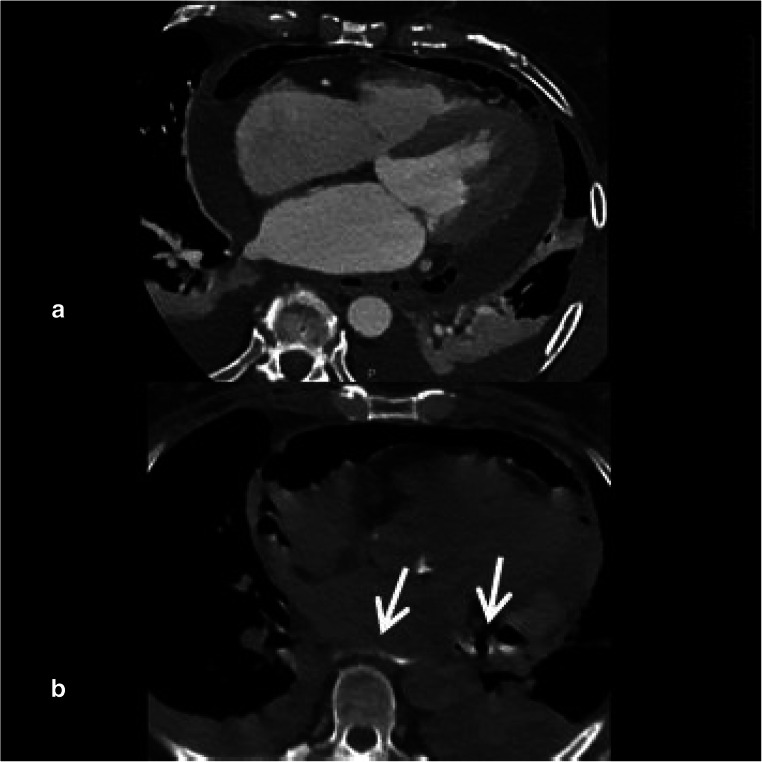
Panel **a**: Left atrial CT angiography rules out atrio-esophageal fistula as neither air bubble in the cardiac chambers nor contrast extravasation is visible. On the other hand, pneumopericardium is present. Panel **b**: Swallow test with gastrografin under CT imaging shows that the contrast agent enters the pericardium (arrows) but does not enter the left atrium; thus, diagnosis is a pericardial-esophageal fistula

A 68-year-old female (BMI 33.2 kg/m^2^) underwent pulmonary vein isolation with a contact force-sensing ablation catheter (power 30 W, temperature limit 43 °C, target force-time integral 350–450 gs) in conscious sedation. No esophageal device was used as per institutional protocol, and no proton pump inhibitor was administered. Three days later, she was admitted to a county hospital due to small pericardial effusion, odynophagia, and subfebrility, without neurological symptoms. Chest CT was negative. Drug treatment of pericarditis was started. Twelve days after the ablation, the patient was referred and admitted to our hospital due to significant pericardial effusion. A purulent fluid was drained via urgent pericardiocentesis on the same day. A few hours later, left atrial CT angiography and swallow test with gastrografin revealed pericardial-esophageal fistula (PEF) (Fig. 1) [1, 2]. An urgent operation was scheduled for the next morning, involving both a heart surgeon and a esophagus surgeon, with the backup of extracorporeal circulatory support (ECS). During surgery, removal of the injured part of the esophagus caused a massive left atrial bleeding indicating atrio-esophageal fistula (AEF) (localized at the posterior antrum of left-sided pulmonary veins), mimicked by the adhesion of inflamed tissue. ECS was initiated, and surgeons were able to reconstruct the damaged tissues, but after the very severe bleeding, the metabolic status of the patient showed progressive deterioration despite the presence of ECS. The weaning from the ECS was unsuccessful despite three attempts, and the patient died on the table.

This is the first report of an atrio-esophageal fistula that was presented as a pericardial-esophageal fistula. Although literature data are controversial, the use of an esophageal device might be reasonable, and post-procedural administration of proton pump inhibitors might be useful to reduce the possibility of AEF. Literature data are controversial on the use of ECS, as smaller fistulas could be resolved off-pump, as well. However, as the size of the lesion is always unknown before the operation, it should be scheduled with the presence of a heart surgeon and esophagus surgeon and with the use of an ECS system [3]. Our report indicates that the operating team has to be prepared for the potential presence of an AEF, even if the imaging modalities suggest PEF. Based on our case, PEF and AEF should be considered a continuum and not as separate entities. Unfortunately, the mortality rate remains high even in case of quick diagnosis and appropriate surgical preparation.
